# Comparison of the Abdominal Wall Muscle Thickness in Female Rugby Players Versus Non-Athletic Women: A Cross-Sectional Study

**DOI:** 10.3390/medicina56010008

**Published:** 2019-12-25

**Authors:** Vanesa Abuín-Porras, Mónica de la Cueva-Reguera, Pedro Benavides-Morales, Rocío Ávila-Pérez, Blanca de la Cruz-Torres, Helios Pareja-Galeano, María Blanco-Morales, Carlos Romero-Morales

**Affiliations:** 1 Faculty of Sport Sciences, Universidad Europea de Madrid, Villaviciosa de Odón, 28670 Madrid, Spain; monica.delacueva@universidadeuropea.es (M.d.l.C.-R.); pbenavidesmorales@yahoo.es (P.B.-M.); fisioroavila@hotmail.com (R.Á.-P.); helios.pareja@universidadeuropea.es (H.P.-G.); maria.blanco@universidadeuropea.es (M.B.-M.); carlos.romero@universidadeuropea.es (C.R.-M.); 2 Department of Physiotherapy, University of Seville, Avicena Street, 41011 Sevilla, Spain; bcruz@us.es

**Keywords:** ultrasound imaging, rugby players, oblique abdominals, transversus abdominis, rectus abdominis, female athletes, pelvic floor disorders, urinary incontinence

## Abstract

*Background and Objectives*: Rugby players engage in demanding, high loading muscular activity in the spine. Study of the abdominal wall architecture in female rugby athletes is relevant to the possible muscular asymmetry secondary to sport practice and the relationship between the abdominal wall and the pelvic floor muscles. Activation of the transversus abdominis (TrAb) generates an increase in the bladder neck muscle. Moreover, an increased interrecti distance (IRD) is related to urinary incontinence and has a higher prevalence in athletic women. The aim of the present study was to compare and quantify, with ultrasound imaging (USI), the thickness of the transversus abdominis (TrAb), external oblique (EO), internal oblique (IO), rectus abdominis (RA), and interrecti distance (IRD) in female rugby players versus non-athletic women in order to improve upon existing knowledge about abdominal wall configuration in female athletes. *Materials and Methods*: A sample of 32 women was recruited at the Universidad Europea Research Lab and divided in two groups: a rugby group (*n* = 16) and a non-athletic women group (*n* = 16). The thickness of the TrAb, EO, IO, RA, and IRD were assessed by USI in both groups. *Results*: There were statistically significant differences for the ultrasound evaluation thickness of the right TrAb (*p* = 0.011; *d* = 0.10), EO (*p* = 0.045; *d* = 0.74), IO (*p* = 0.003; *d* = 1.32), and RA (*p* = 0.001; *d* = 1.38) showing a thickness increase for the rugby group with respect to the control group. For the IRD thickness, there were no significant differences (*p* > 0.05) between groups. *Conclusions*: An increased TrAb, IO, EO, and RA thickness may be shown in female rugby players versus non-athletic women. Nevertheless, statistically relevant differences were not found for the IRD between both groups.

## 1. Introduction

The structural integrity of the abdominal wall is provided by the muscular, fascial, and connective tissue layers, ensuring the protection of the abdominal viscera and spine [1,2]. In addition, this abdominal wall integrity is also necessary to stabilize the trunk and provide the capability for functional movements [3]. Abdominal muscles present a paired disposition; the external oblique (EO), internal oblique (IO), and transversus abdominis (TrAb), in both sides of the trunk, and in the midline, the rectus abdominis (RA). Abdominal wall muscles work in a coordinated manner with the diaphragm and the pelvic floor muscles, transferring loads around the trunk and increasing the abdominal pressure, a mechanism needed for bowel movements, urination, and childbirth [4,5].

The use of ultrasound imaging (USI) has been exponentially increasing and was described as a non-invasive, safe, and non-time-consuming tool to assess soft tissue features, such as thickness, length, cross-sectional area (CSA), and morphology [6,7]. In addition, different structures have been assessed, including the pelvic floor [8]. Research with USI has allowed the assessment and quantification of different muscle structures, such as the supra and infraspinatus muscles [9], femoral quadriceps [10], brachial biceps [11], TrAb and IO [12], rectus abdominis [13], multifidus [14], diaphragm [15], trapezius [16], peroneus [17], extrinsic foot muscles [18], intrinsic plantar muscles [19,20], rectus femoris, and sartorius and iliopsoas [21].

Computerized axial tomography [22] and magnetic resonance imaging (MRI) [23] were considered as the gold standard for abdominal wall examination. Nevertheless, USI may be considered as a highly reliable alternative methodology [1,23], being reported as a valid tool to assess the distance between the rectus abdominis muscles at rest and during contraction [24,25]. Furthermore, muscle thickness measured by USI presents a high correlation with CSA values measured with MRI in subjects with different training levels [26].

USI assessment of the abdominal wall has shown excellent intra- (ICC 0.92–0.99) [1] and inter-rater (ICC 0.91–0.99) [27] reliability values. In addition, several authors reported that the interrecti distance (IRD) assessment was a valid and reliable measure by USI in healthy individuals [24,25].

The USI examination of the abdominal wall muscles has been previously carried out in healthy populations [28,29], postpartum women [30] and several sport disciplines, such as baseball [26], handball [31], cricket [32], and female football players [33]. Moreover, differences in abdominal wall features were reported according to different levels of activity in basketball [34], tennis [35], and Pilates [36].

Regarding the abdominal wall muscle activity implications in sports training and competition, rugby players engage in demanding, high-loading muscular activity in the spine during the launching, reception, and blocking movements [37]. Several studies reported the importance of the core muscles assessment, as the inner unit along with the pelvic floor muscles has begun to be employed in therapeutic approaches for not only urinary incontinence (UI), but also lumbar and lumbopelvic pain [38,39].

The execution of asymmetric repeated actions in the rugby context could influence the morphology of the abdominal muscles [40]. Therefore, more studies are needed to establish the relationship between injury risk and the asymmetry of the muscular architecture [35].

Several studies have evaluated the IRD in women by USI evaluations [41,42], due to the implications of abdominis rectus diastasis in the pelvic floor muscle function and urinary incontinence [43]. Recent research suggests that sports practice increases the risk and prevalence of UI and that the type of sport activity performed by women also influences the development of the pathology [44].

To date, no previous studies have examined the features of abdominal wall muscle morphology in female rugby players. The purpose of the present study was to compare and quantify with USI the thickness of the TrAb, EO, IO, RA, and IRD in female rugby players versus non-athletic women. We hypothesized that abdominal wall muscles thickness was increased in the rugby players secondary to the prolonged mechanical loads exposure during training and competition.

## 2. Materials and Methods

### 2.1. Design

A case–control study was developed from February to July 2019, following the guidelines of the Strengthening the Reporting of Observational Studies in Epidemiology Statement (STROBE) guidelines [45].

### 2.2. Ethics

The study was approved by the Intervention Clinical Committee of the European University of Madrid, Spain (CIPI/19/003; 10 February 2019). The present study was adhered to the ethical standards of the Declaration of Helsinki. In addition, the consent inform form was obtained from all subjects before the beginning of the study.

### 2.3. Subjects

A total sample of 32 women was recruited at the Universidad Europea Research Lab and divided into two groups: semi-professional players from an entertainment Spanish division for the rugby group (*n* = 16, aged 24.73 ± 4.90 years) and healthy women who do not practice any sport activity for the non-athletic women group (*n* = 16, aged 27.93 ± 6.13 years). The inclusion criteria were subjects from 18 to 45 years [10,29], female gender, and non-pregnant and nulliparous women. The exclusion criteria were a body mass index (BMI) greater than 31 kg/m^2^ [34], previous abdominal surgery or abdominal hernia [29], active rheumatologically disorders or connective tissue alterations [46], systemic diseases [28], neurological disorders, neuromuscular and/or respiratory pathology [28,47], and orthopedic surgical procedures in lumbar, pelvic, or lower limbs during the previous 6 months. Moreover, skin diseases of the abdominal region [28] and allergic reactions to the ultrasound gel were considered as exclusion criteria [48].

For the sample size calculation G*Power software for iOS was used with the difference between two independent means based on the difference between rugby and control groups using the TrA thickness (mm) variable of a pilot study (*n* = 16), divided into eight individuals for the rugby group (0.45 ± 0.11) and eight individuals for the control group (0.33 ± 0.09). A power of 0.80, an α error of 0.05 and an effect size of 1.19 with two-tailed hypotheses were used. To conclude, a sample of 26 participants was estimated. Considering a possible 20% loss to follow-up, we recruited a sample of 32 participants.

### 2.4. Outcome Measures

**Figure 1:**
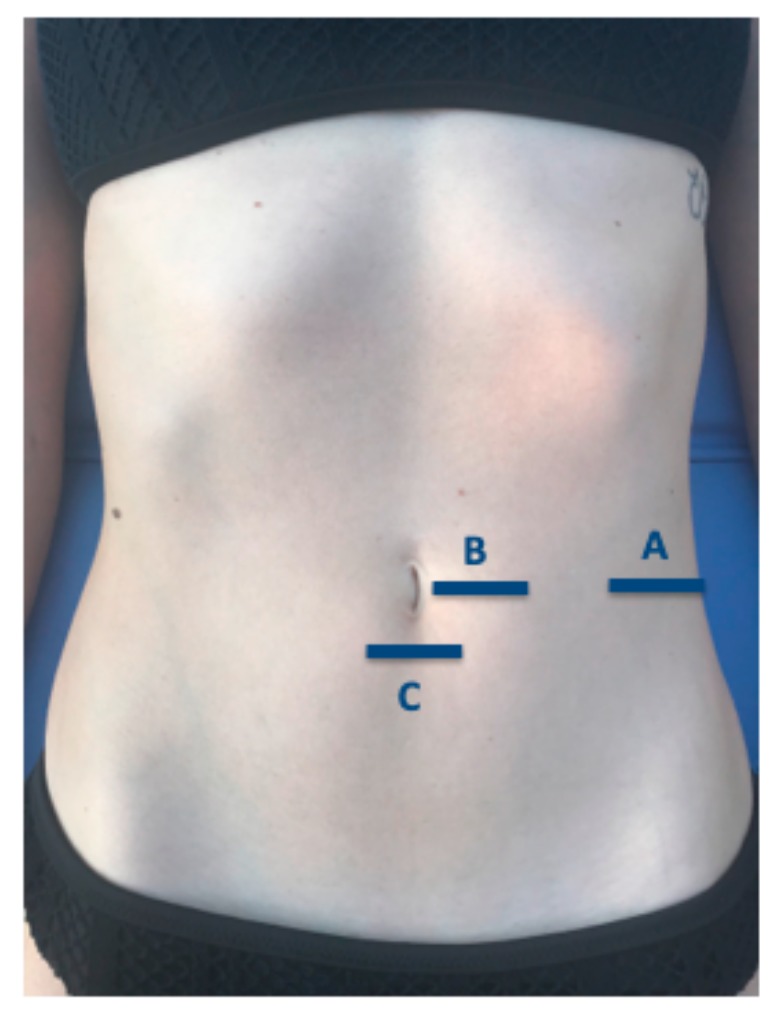
Probe locations during ultrasound evaluation of the abdominal wall muscles.

**Figure 2:**
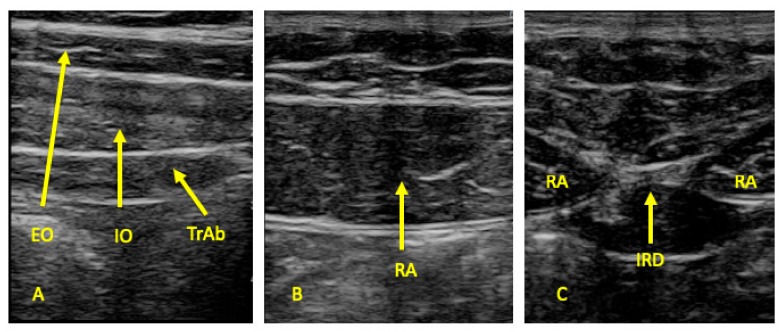
Muscle thickness and IRD measurements of the abdominal wall muscles. Abbreviations: EO, external oblique; IO, internal oblique; IRD, interrecti distance; TrAb, transversus abdominis; RA, rectus abdominis. (**A**): EO, IO, TrAb images. (**B**): RA images. (**C**): IRD images.

All measures were carried out by the same therapist (P.B.M) with USI experience. An ultrasound system (LOGIC S7, XDclear, GE Healthcare; Little Chalfont, UK) with a 10–13 MHz range linear transducer (with 55 mm footprint) was used in B mode for ultrasound imaging measurements. All participants were placed in supine position for the ultrasound data collection. Following the Whittaker et al. [47] guidelines, ultrasound images of the EO, IO, and TrAb muscles were performed by placing the transductor in the mid-axillary line, halfway between the subcostal line and the iliac crest (Figure 1A and Figure 2A). For the RA examination, the probe was placed aligned with the umbilicus, and just under the umbilicus for the IRD measurement (Figure 1B and Figure 2B). Whittaker et al. [23] argued that IRD was considered the horizontal distance between the both RA muscles (Figure 1C and Figure 2C). Muscle thickness was described as the distance between the edges of each muscle border. In addition, following prior studies about abdominal wall muscles examined by USI, all the muscle measurements were conducted excluding the fascial and perimuscular connective tissues [23,49,50].

The mean of three repeated values was recorded for each measurement at the end of expiration, maintaining the transducer at the same place and with the same pressure (pressure generated by the weight of the transducer). All the measurements were evaluated at the right side. ImageJ software (Research Services Branch, National Institute of Mental Health, Bethesda, MD, USA) was employed to measure all the images offline [49]. The operator who analyzed the images using ImageJ software was blinded due to the patients being codified prior to measuring the images offline.

### 2.5. Statistics Analysis

Statistical analysis was performed with the Statistical Package for Social Sciences (SPSS) for iOS (v.22, IBM, Armonk, NY, USA). First, the Shapiro–Wilk test was employed to assess the normality. Second, the descriptive analysis for the total sample was carried out. Finally, a comparative analysis for both groups was performed. For parametric data, the mean, standard deviation (SD), and Student’s *t*-test for independent samples were used for data analysis. In addition, Levene’s test was utilized to check the equality of variances. The median, interquartile range (IR), and Mann–Whitney *U* test were applied for non-parametric data analysis. The effect size between groups was estimated through the use of Cohens’s *d*. To interpret the effect size results, Cohen suggested that *d* = 0.02 was considered “small”, *d* = 0.5 was considered “medium” and a *d* = 0.08 was considered a “large” effect size [50]. For all statistical tests, an α error of 0.05 (95% confidence interval) and a desired power of 80% (β error of 0.2) were used.

## 3. Results

The age, weight, height, and BMI of the sample were homogeneous between the rugby and control groups (Table 1). There were statistically significant differences for the ultrasound evaluation thickness of the right TrAb (*p* = 0.011; *d* = 0.10), EO (*p* = 0.045; *d* = 0.74), IO (*p* = 0.003; *d* = 1.32), and RA (*p* = 0.001; *d* = 1.38) showing a thickness increase for the rugby group with respect to the control group. For the IRD thickness, there was no significant differences (*p* > 0.05) between groups (Table 2; Table S1).

## 4. Discussion

To our knowledge, this is the first study performing a comparison of the abdominal wall muscles between female rugby players and non-athletic women. Previous studies explored muscle thickness measures at rest in order to establish reference values [28,29]. The results of the present study are coincident with prior studies that assessed and quantified the abdominal wall muscles. For example, Rankin et al. [28] established the normal reference ranges for abdominal muscle size and symmetry in healthy subjects in order to enable comparison with other clinical groups and populations. Teyhen et al. [51] reported changes in TrAb and IO muscle thickness in healthy individuals who performed six common trunk strengthening exercises. Beer et al. [29] in a study carried out in 150 nulliparous women, detected the location by USI and the morphological features of the linea alba and the IRD.

Several authors have shown differences in the muscle thickness in basketball [34] and baseball [30] players compared to non-athletic subjects. In addition, Kubo et al. [52] reported a thickness increase of the abdominal wall muscles for elite football players in relation to amateur football players.

Regarding the muscular asymmetries between dominant and non-dominant sides, several authors reported differences in the abdominal wall complex. For example, Jones et al. [37] observed a thickness increase in the non-dominant side in cricket players. Based on these findings, it is possible to justify the muscle thickness increase in the non-dominant side with the repeated and powerful trunk rotations of the dominant hand in the hit and launch movements. Several authors observed a RA thickness increase for the non-dominant side with USI and MRI assessments in tennis players [35,53,54]. Although in the present study only the right side was evaluated, authors argued that the side-to-side asymmetry in muscle thickness founded in prior studies could be explained by the repetitive reception movements carried out during training and competitions [55]. Clinical implications of this muscular asymmetry in the trunk still need to be explored.

Whittaker et al. showed significant changes in RA thickness in patients with lumbar pain, with wider IRD and changes in the perimuscular connective tissue [1]. This may have implications in the injury prevention field in female rugby players through the USI examination of their abdominal wall muscular architecture.

IRD showed no statistically relevant differences in both groups in this study. An increased IRD distance is related to pelvic floor disorders and urinary incontinence [42], which has a higher prevalence in athletic women compared to sedentary populations [56]. IRD in women is principally altered during postpartum [41]. Therefore, the fact that women from our sample were nulliparous could explain the results.

Moreover, the increased TrAb thickness found in this study may be considered as a sign of continuous activation of this muscle during training and practice of this sport. There is also a close relationship between the musculature of the pelvic floor and the abdominal musculature [57]. An activation of the TrAb generates an increase in the bladder neck muscle (continence mechanism) [58]. Therefore, the increased thickness due to frequent activation of the TrAb in rugby practice may be considered as a protective factor for urinary incontinence in female athletes.

### 4.1. Clinical Implications

The findings of the present study did not provide a cause or explanation about the pelvic floor disorders in female athletes populations. We suggest that the examination of the abdominal wall muscles by USI could help to develop a complete evaluation for the prevention and managing the lumbopelvic disturbances that occur in female athlete populations. The direct relationship between core muscles and pelvic floor muscles and the good reliability for assessing the abdominal muscle thickness by USI present an interesting evaluation approach for researchers and clinicians.

### 4.2. Limitations and Future Lines

Several limitations in this study should be considered. First, all assessments have been carried out only at rest. It is possible that changes during specific muscle actions could provide information of interest for sports training [51]. In addition, Lee and Hodges [59] argued that IRD could be modified when the subjects were performing training exercises and were exposed to external loads. Second, rugby players with conditions such as low back pain or lumbopelvic pain were not studied, and this could be useful in future research. Third, following the Romero et al. [18] procedure, the evaluator who recorded the USI images was not blinded. However, the operator who analyzed the images using ImageJ software was blinded due to the patients being codified prior to measuring the images offline. Finally, the present study only compares rugby players with non-athletic women. Further research with a larger sample is needed to compare aspects such as game position and other training modalities, which would lead to a deeper understanding of the training needs of female rugby players in order to enhance performance and promote injury prevention.

## 5. Conclusions

In conclusion, an increased TrAb, IO, EO, and RA thickness may be shown in female rugby players versus non-athletic women. However, statistically relevant differences were not found for the IRD between both groups.

## Figures and Tables

**Table 1:** Sociodemographic data of the sample.

Data	Rugby (*n* = 16)	Controls (*n* = 16)	*p*-Value Cases vs. Controls
Age, y	24.73 (4.90) *	27.93 (6.13) *	0.127 **
Weight, kg	61.0 (60.0–69.0) ^†^	61.50 (53.0–70.0) ^†^	0.285 ^‡^
Height, m	1.65 (1.63–1.69) ^†^	1.64 ± (1.58–1.69) ^†^	0.389 ^‡^
BMI, kg/m^2^	23.44 (21.9–24.7) ^†^	22.79 (20.5–25.7) ^†^	0.412 ^‡^

Abbreviations: VAS, visual analogue scale; body mass index (BMI). * Mean (standard deviation) was applied. ** Student´s *t*-test for independent samples was performed. ^†^ Median (25th percentile, 75th percentile) was used. ^‡^ Mann–Whitney *U* test was utilized.

**Table 2:** Ultrasound imaging of the abdominal wall muscles.

Measurement	Rugby (*n* = 16)	Controls (*n* = 16)	*p*-Value
Distance (cm)			
IRD	0.44 (0.13) *	0.44 (0.37–0.60) ^†^	0.367 ^‡^
Thickness (cm)			
Right TrAb	0.41 (1.06–1.03) ^†^	0.33 (0.07) *	0.011 ^‡^
Right IO	0.93 ± 0.07 *	0.80 ± 0.12 *	0.003 **
Right EO	0.73 ± 0.13 *	0.64 ± 0.11 *	0.045 **
Right RA	1.19 ± 0.12 *	1.03 ± 0.11 *	0.001 **

Abbreviations: EO, external oblique; IO, internal oblique; IRD, interrecti distance; RA, rectus anterior; TrAb, transversus abdominis.* Mean (standard deviation) was applied. ** Student´s *t*-test for independent samples was performed. ^†^ Median (25th percentile, 75th percentile) was used. ^‡^ Mann–Whitney *U* test was utilized.

## Supplementary Materials

The following are available online at https://www.mdpi.com/1010-660X/56/1/8/s1, Table S1: Effect size between groups.
