# Supplementary material for: Comparison of the Abdominal Wall Muscle Thickness in Female Rugby Players Versus Non-Athletic Women: A Cross-Sectional Study
Source: Medicina (Kaunas). 2019 Dec 25;56(1):8. doi: 10.3390/medicina56010008 (PMC7022579; doi:10.3390/medicina56010008)
Supplement: Supplementary file 1 [file medicina-56-00008-s001.pdf]

Supplementary material

Table S1. Effect size between groups.

Measurement	Rugby (n = 16)	Controls (n = 16)	p-value (Cohen´s d effect size)
Distance (cm)			
IRD	0.44 (0.13) *	0.44 (0.37–0.60) †	0.367 ‡ (0.00)
Thickness (cm)			
Right TrAb	0.41 (1.06–1.03) †	0.33 (0.07) *	0.011 ‡ (0.10)
Right IO	0.93 ± 0.07 *	0.80 ± 0.12 *	0.003 ** (1.32)
Right EO	0.73 ± 0.13 *	0.64 ± 0.11 *	0.045 ** (0.74)
Right RA	1.19 ± 0.12 *	1.03 ± 0.11 *	0.001 ** (1.38)

Abbreviations: EO, external oblique; IO, internal oblique; IRD, interrecti distance; RA, rectus anterior; TrAb, transversus abdominis. * Mean (standard deviation) was applied. ** Student´s t-test for independent samples was performed. † Median (25th percentile, 75the percentile) was used. ‡ Mann-Whitney U test was utilized.
